# Formation and Growth of Intermetallic Compounds during Reactions between Liquid Gallium and Solid Nickel

**DOI:** 10.3390/ma14195694

**Published:** 2021-09-30

**Authors:** Doyoung Lee, Chang-Lae Kim, Yoonchul Sohn

**Affiliations:** 1 Department of Welding and Joining Science Engineering, Chosun University, Gwangju 61452, Korea; ldy6744@naver.com; 2 Department of Mechanical Engineering, Chosun University, Gwangju 61452, Korea; kimcl@chosun.ac.kr

**Keywords:** gallium, intermetallics, interfacial reaction, X-ray diffraction, scanning electron microscopy

## Abstract

Liquid metals, such as Ga and eutectic Ga-In, have been extensively studied for various applications, including flexible and wearable devices. For applying liquid metal to electronic devices, interconnection with the various metal electrodes currently in use, and verifying their mechanical reliability are essential. Here, detailed investigations of the formation and growth of intermetallic compounds (IMCs) during the reactions between liquid Ga and solid nickel were conducted. Ga and Ni were reacted at 250, 300, and 350 °C for 10–240 min. The IMC double layer observed after the reactions contained a Ga_7_Ni_3_ bottom layer formed during the reactions, and a Ga_x_Ni top layer (with 89–95 at.% of Ga) precipitated during cooling. Numerous empty channels exist between the rod-type Ga_7_Ni_3_ IMCs. Ga_7_Ni_3_ growth occurred only in the vertical direction, without lateral coarsening and merging between the rods. The time exponents were measured at 1.1–1.5, implying that the reaction kinetics were near-interface reaction-controlled. The activation energy for Ga_7_Ni_3_ growth was determined as 49.1 kJ/mol. The experimental results of the Ga-Ni reaction study are expected to provide important information for incorporating liquid metals into electronic devices in the future.

## 1. Introduction

Liquid Ga- and Ga-based alloys, such as eutectic Ga-In and galinstan [1,2], have attracted widespread attention, because of their potential properties of deformability, miniaturization, low process temperature during fabrication, and low toxicity. The growing demand for flexible and wearable devices, including prosthetics and implantable devices, has inspired the research, and led to an increase in the production of deformable electronic devices [3,4]. Many promising circuits [5,6,7] and electronic components [8,9,10] incorporating liquid metals, which can be bent or stretched, have been recently demonstrated. In addition, basic processing techniques for liquid metals have been investigated, in order to realize their potential applications. These efforts include injecting and printing liquid alloys [11,12,13] into various microfluidic channels or substrates.

To construct an electronic device, the chip and substrate must be electrically connected. Soldering is a fundamental interconnection technology used in microelectronic packaging. There have been ongoing efforts to formulate various alloying elements with Sn-based solders, such as Sn-3.0Ag-0.5Cu and Sn-58Bi, to create alloys that enable low-temperature soldering processes [14]. Such alloys would reduce energy consumption, and the risk of damaging components. Owing to their low melting points, and the ability to form intermetallic compounds (IMCs) with various metals, Ga and Ga-based alloys have been used as potential soldering materials in microelectronics. However, such studies have mainly focused on reactions with Cu, and reactions with other metals have rarely been studied.

The reports on the reaction between Ga and Al demonstrate that Ga has a low solubility in Al, does not form IMCs, and penetrates along the Al grain boundary during the reaction, forming channels ranging in thickness from several nanometers to several micrometers [15,16]. In the case of Cu, since the solid solubility of Ga is large and it forms a Cu-Ga IMC, there are few reports on the embrittlement phenomenon. It is mainly studies on low-temperature junctions for electronic packaging applications that have been reported [17,18]. S.K. Lin et al. [17] conducted a reaction study of Cu/Ga and Cu/Ga/Cu diffusion couples. Cu atoms were preferentially dissolved into the exposed grain boundaries, generating a basin-type consumption of the Cu substrate. They observed two IMCs of Cu_9_Ga_4_ and CuGa_2_, and determined time exponents of 1.0 at 160 °C and ~2.0 for 200–240 °C. In the work of Lin et al. [18], liquid Ga reacted with polycrystalline Cu, single-crystal Cu, and Pt/single-crystal Cu. They observed that, in polycrystalline Cu, a non-uniform interface was formed owing to Ga penetration, whereas in single-crystal Cu, a less non-uniform interface was formed. Furthermore, a completely flat interface was obtained when the Pt protective layer was coated. Interfacial reaction studies of Ga/Ni have rarely been conducted. Chen et al. [19] conducted a reaction study with Cu/Ga/Ni diffusion couples and found a Ga_7_Ni_3_ phase at 200–350 °C and Ga_3_Ni_2_ at 500 °C. They reported that the Ga consumption depended linearly on the square root of the reaction time. Another study proved that the Cu/Ni/Ga/Ni/Cu reaction couples [20] could fully transform into Cu/face-centered cubic (fcc)-(Ni, Cu, Ga)/Cu solid-solution joints. During the reaction between Ga and Ni, Ga_7_Ni_3_ was determined to be the major forming phase at 300 °C, with a time exponent of ~1.0. Experimental results of previous studies are summarized in Table 1, as a comparison to the results of this study. However, the growth mechanism of Ga-Ni IMC is not completely understood, and detailed kinetic studies have not yet been reported. In this study, a detailed investigation of the interfacial reaction between liquid Ga and solid Ni was conducted in the temperature range of 250–350 °C. Below 200 °C, IMC was formed only in some areas, not on the entire Ni substrate, and the IMC growth rate was very slow, making it unsuitable for IMC growth behavior studies. The morphology and growth mechanisms of the Ga-Ni IMCs were elucidated, along with the time exponents for their growth kinetics. In general, the reaction with Ni is known to be relatively slow compared to the reaction with Cu, and the purpose of this study is to provide accurate quantitative data on the Ga-Ni reaction. The experimental results provide fundamental implications for Ga-Ni interconnections for various electronic applications.

## 2. Materials and Methods

### 2.1. Materials and Specimen Preparation

**Figure 1:**
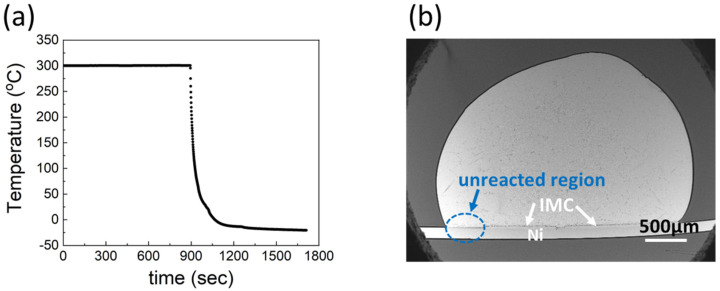
(**a**) Example of heat treatment process for the Ga-Ni interfacial reaction, (**b**) reaction product after the interfacial reaction at 300 °C for 60 min.

Ga metal (99.999%) and Ni foil (99.994%, 0.1 mm thick) were purchased from Alfa Aesar. The surface of Ni was cleaned, and etched with a mild HCl solution to remove surface oxides and improve the wettability. To fabricate the specimens for interfacial reactions between Ga and Ni, 0.05 g of Ga metal was dropped on a 0.1 mm thick Ni foil with dimensions of 5 mm × 10 mm, and reacted at temperatures of 250, 300, and 350 °C, respectively. The interfacial reactions were conducted in a convection oven for 10–240 min. After the reactions, the specimens were cooled in air for 2 min, followed by solidification in a freezer for over 30 min at −20 °C. An example of heat treatment process is presented in Figure 1a. The specimens were then mounted in epoxy, ground, and polished in order to examine their cross-sections. Ga etching was conducted using a mild HCl solution (10 vol.%, in DI water), to clearly distinguish the IMCs from the unreacted Ga. A reaction product is presented in Figure 1b. The Ni substrates did not completely react with Ga at 250 and 300 °C. For example, no IMC was observed on the left-hand side of the specimen reacted at 300 °C for 60 min, as shown in the circled area in the figure.

### 2.2. Characterization

Cross-sectional micrographs of the specimens were acquired using scanning electron microscopy (SEM). In addition, the IMCs were identified using energy dispersive X-ray (EDX) analysis and electron microprobe analysis (EPMA). The thickness of the IMC in the scanned micrographs was measured using an image analysis software. The thickness of the layer was defined as the total area occupied by the phase divided by the length. Average values were obtained after measuring five different regions for each reaction specimen. The unreacted areas on the Ni substrate were excluded from the measurements. Further, accurate phase identification of the interfacial IMCs was conducted using X-ray diffraction (XRD). For fabricating the XRD specimens, the interfacial IMCs on the Ni surface were revealed by completely etching the unreacted Ga after the Ga-Ni interfacial reactions.

## 3. Results

### 3.1. Identification of the Ga-Ni Interfacial IMCs

**Figure 2:**
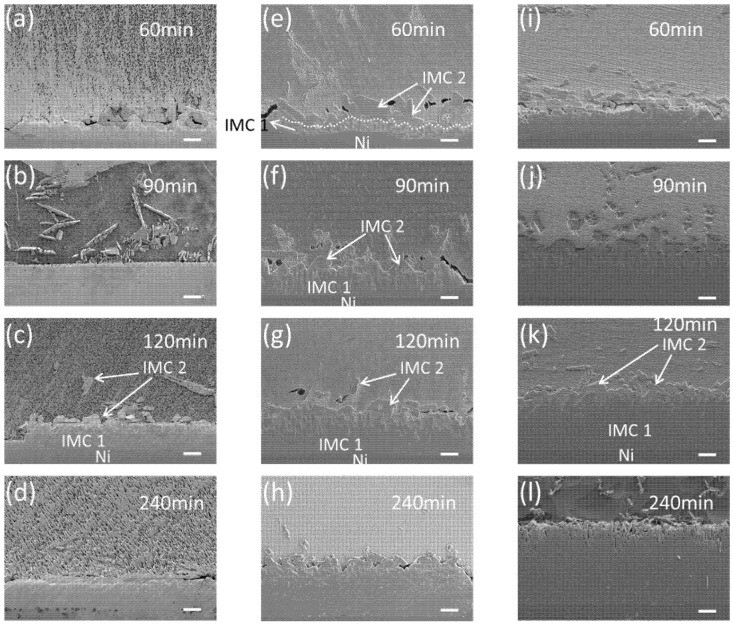
SEM micrographs of the reaction products for Ga-Ni reactions at: (**a**–**d**) 250 °C, (**e**–**h**) 300 °C, (**i**–**l**) 350 °C. (scale bar: 20 µm).

**Figure 3:**
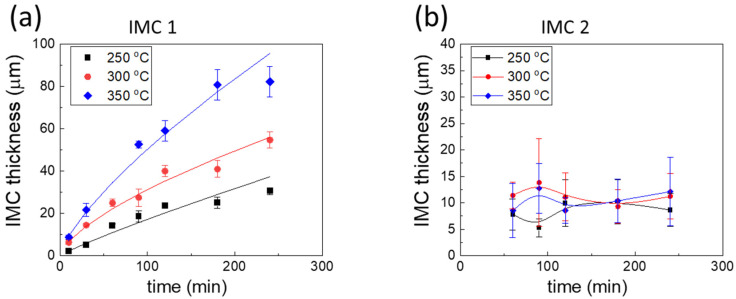
Measured thicknesses of the (**a**) IMC underlayer and (**b**) IMC overlayer at the reaction interfaces as a function of the reaction time.

Reactions between Ga and Ni were conducted at temperatures of 250, 300, and 350 °C, and the reaction products are presented in Figure 2. Two layers of IMCs were observed after the reactions. The bottom layer (IMC 1) exhibits a greater contrast than the top layer (IMC 2). The consumption of the Ni substrate was not completely uniform. Irregular consumption was observed in some regions of the reaction specimens, as shown in Figure 2e. In addition, some parts of the Ni substrate did not react with Ga at 250 and 300 °C, as shown on the left-hand side of Figure 2c, although the unreacted region was absent in the specimens reacted at 350 °C. For all reaction temperatures, IMC 1 grew continuously with the increasing reaction time. After a long reaction time, the bottom of IMC 1 showed layer-type morphology, while the top side consisted of a large number of individual grains, with some spaces between them. These spaces were completely filled with IMC 2. IMC 2 formed the upper layer of the interfacial reactants, and showed a similar thickness in all specimens; this was not the case for IMC 1. IMC2 was observed not only on the top of the reaction interface, but also on the inside of unreacted Ga after the reactions. The thicknesses of the top and bottom IMCs were measured, and are plotted in Figure 3a,b, respectively. The thickness of IMC 1 increased with the reaction time. The growth rate of IMC 1 was approximately linearly dependent on the reaction time, indicating the rapid reaction rate and the faster growth rate of the IMC, compared to those of the Cu-Ga IMCs formed at 200–300 °C [17]. Meanwhile, the thickness of IMC 2 was similar for all specimens, regardless of the reaction temperature and time. The thicknesses ranged between 5 μm and 15 μm, with an average value of ~10 μm. A detailed analysis of the IMC growth mechanism is presented in the next section.

**Figure 4:**
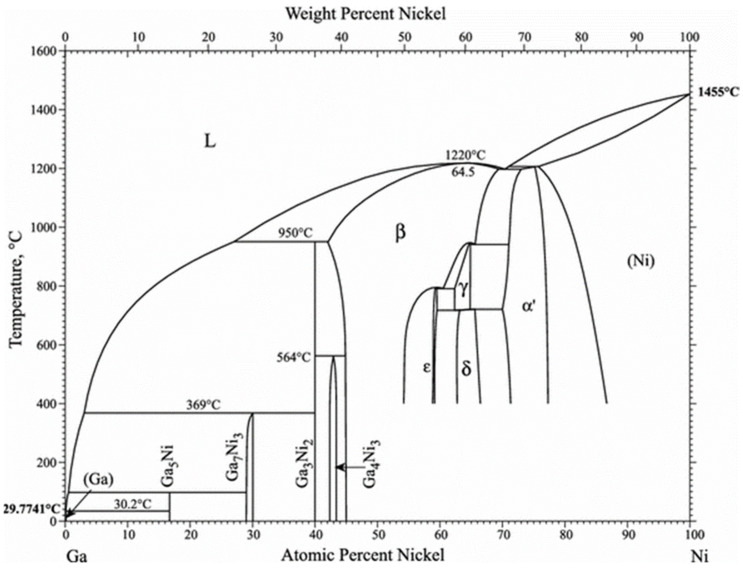
Binary phase diagram of Ga-Ni [21].

**Figure 5:**
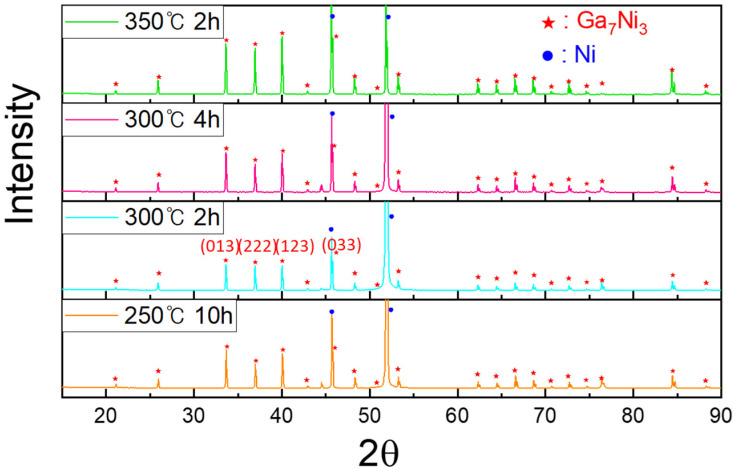
X-ray diffraction data for the IMC formed at the Ga/Ni reaction interfaces. Each specimen was prepared after the complete etching of unreacted Ga.

**Figure 6:**
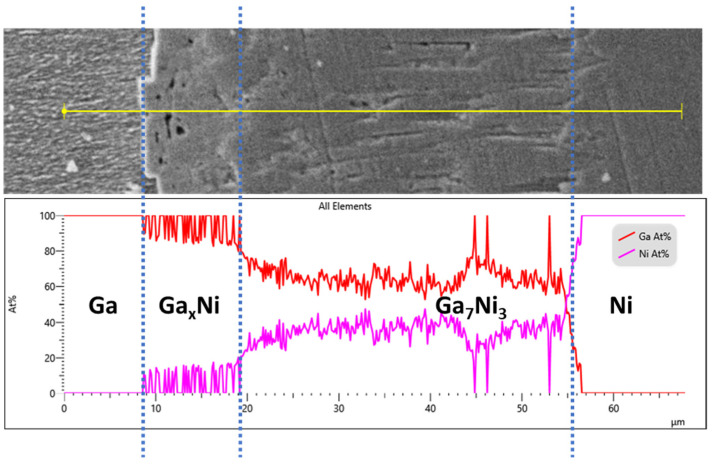
EPMA line scan of Ga/Ni specimens reacted at 300 °C for 180 min.

**Figure 7:**
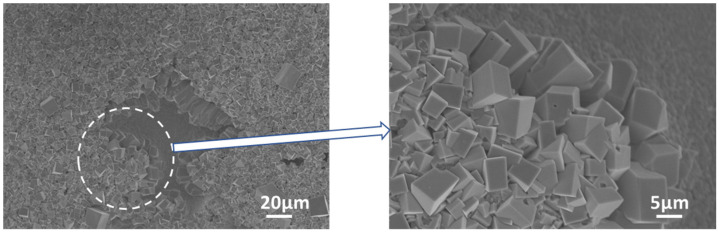
Plane-view SEM micrographs of Ga_x_Ni IMCs in the specimen reacted at 350 °C for 120 min.

The Ga-Ni binary phase diagram presented in Figure 4 shows that four different IMCs can be formed during the reaction between Ga and Ni: Ga_5_Ni, Ga_7_Ni_3_, Ga_3_Ni_2_, and Ga_4_Ni_3_. The final version of the Ga-Ni phase diagram was constructed using the modified Ga-rich part for 0–48 at.% of Ni [21] and the work of Okamoto for the remaining diagram [22]. The specimens that reacted at each reaction temperature were analyzed, using XRD to identify the phases formed at the reaction interfaces. The remaining unreacted Ga was completely etched overnight, using the etching solution. The XRD patterns are shown in Figure 5. Only one phase (IMC 1) was detected in all the specimens. The bottom layer, IMC 1, was found to be the Ga_7_Ni_3_ phase, which showed a polycrystalline microstructure with the major planes (013), (222), (123), and (033), planes without a specific preferred orientation. As mentioned earlier, IMC 1 grew with increasing reaction time, so it could be seen that the strength of the XRD peaks increased as the reaction time at 300 °C increased from 2 h to 4 h. In addition, it could also be confirmed that, when the reactions were conducted for the same reaction time, a larger amount of IMC 1 was formed at 350 °C than at 300 °C. However, the upper layer, IMC 2, was not detected by XRD analysis, which suggested that the microstructure of IMC 2 may not be perfectly crystalline, but nanocrystalline or amorphous. The IMCs were further analyzed using an EPMA line scan of cross-sectional SEM specimens, as shown in Figure 6. The result showed that IMC 2 contained a larger amount of Ga and less Ni than the Ga_7_Ni_3_ phase. Referring to the phase diagram [21], IMC 2 could be considered the Ga_5_Ni phase. However, the related peaks were not detected in the XRD analysis. Our additional EDX analyses, presented as Supplementary Materials, showed that IMC 2 had about 89–95 at.% of Ga. This was greater than the 83.3 at.% of the Ga_5_Ni phase. Therefore, in this study, IMC 2 could not be determined as Ga_5_Ni and was denoted as Ga_x_Ni. These results also suggest that the Ga_x_Ni phase may be a non-stoichiometric alloy, but additional research is needed to reach an accurate conclusion. Plane-view SEM micrographs of the Ga_x_Ni IMCs are presented in Figure 7. The Ga_x_Ni IMCs are seen to grow in a cuboidal shape. According to the literature [23,24], the Ga-rich compound was difficult to acquire experimentally as a form of single phase, and it was usually associated with the Ga_3_Ni_2_ or Ga_7_Ni_3_ phases. At present, standard XRD spectra of the Ga-rich phase are not found in the inorganic crystal structure database (ICSD).

**Figure 8:**
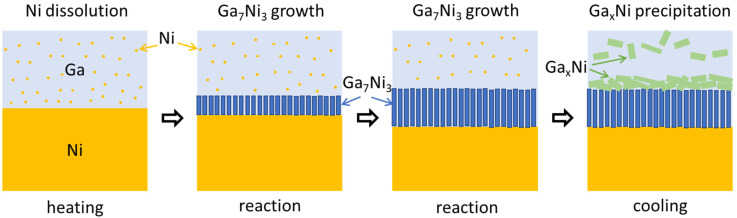
Schematic representation of IMC formation and growth during the reaction between liquid Ga and solid Ni (not to scale).

In Figure 8, we present a schematic diagram for a better understanding of the reaction sequence. When the specimen composed of liquid Ga on a solid Ni substrate was heated, Ni atoms were dissolved into the liquid Ga. From the Ga-Ni phase diagram, the solubilities of Ni in Ga were approximately 1.5, 2.0, and 2.7 at.% for reaction temperatures of 250, 300, and 350 °C, respectively. At the reaction temperatures, the Ga_7_Ni_3_ phase grew at the interface between Ga and Ni. The formation of the Ga_7_Ni_3_ phase has also been reported in the literature, in the temperature range of 200–350 °C [19,20]. Meanwhile, the Ga_3_Ni_2_ phase was reported to form at a higher temperature of 500 °C [19], or at a prolonged reaction time of 3 h at 300 °C [20]. However, this phase was not observed in any specimen fabricated in this study, as shown in Figure 2. The Ga_7_Ni_3_ IMC grew continuously, at the expense of the Ni substrate. After cooling, Ga_x_Ni IMCs were observed on top of the Ga_7_Ni_3_ IMC layer and inside the unreacted Ga. Theoretically, the Ga_7_Ni_3_ phase should also precipitate out from inside the liquid Ga during cooling from the reaction temperature to ~100 °C, because the Ga-rich phase cannot exist over its melting temperature [21]. However, in this study, the Ga_7_Ni_3_ IMCs were found only at the reaction interfaces, and not inside the Ga after the reactions. The rapid cooling rate may be attributed to the scant formation of the Ga_7_Ni_3_ phase inside the Ga. The specimens took less than 1 min to cool down from the reaction temperature to 100 °C, as shown in Figure 1a. Consequently, only the Ga_x_Ni phase precipitated inside the liquid Ga during the cooling process. A study on the Ga-Ni reaction at low temperatures under 200 °C has not yet been reported.

### 3.2. Growth Mechanism of Ga_7_Ni_3_ IMCs

**Figure 9:**
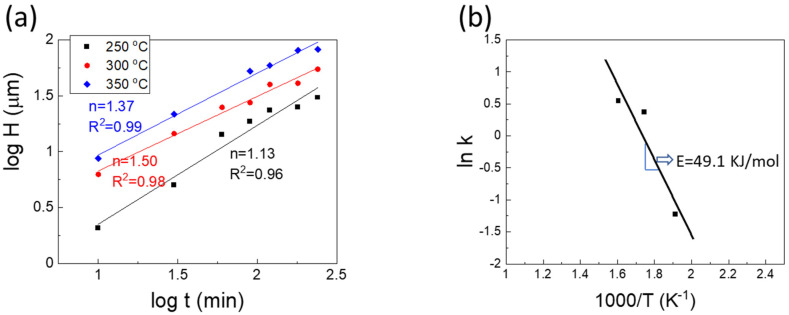
(**a**) Log-log plot of Ga_7_Ni_3_ IMC growth rate with estimated time exponents, (**b**) Arrhenius plot for Ga_7_Ni_3_ IMC growth.

A log–log plot of the interfacial Ga_7_Ni_3_ IMC thickness versus reaction time is presented in Figure 9. The empirical kinetic equation for IMC growth is usually written in the form of Equation (1).

(1) $$H={\mathrm{kt}}^{1/n}=k_{0}\exp\left(-\frac{E}{\mathrm{RT}}\right)t^{1/n}$$

where H, t, T, and E represent the thickness of the IMC, reaction time, temperature, and activation energy, respectively. In addition, k, R, and n represent the kinetic constant, gas constant, and time exponent, respectively. The time exponents calculated from the graph were 1.13, 1.50, and 1.37 for 250, 300, and 350 °C, respectively. However, time exponents close to 1.0 are very rare for the growth of the IMCs formed in the solder joints. Typically, the vertical growth of IMCs, such as Cu_6_Sn_5_ and Ni_3_SN_4_, is usually accompanied by lateral coarsening of the grains. This usually results in a time exponent larger than 2.0, as observed in the case of diffusion-controlled kinetics. Kim et al. [25] explained that the growth of scallop-type Cu_6_Sn_5_ IMCs, assisted by Ostwald ripening, could produce a time exponent of 3.0, which is observed in general diffusion-controlled kinetics. Another theory, which focused on the diffusion through the grain boundaries between the IMC grains, suggested that the kinetics governed by grain boundary diffusion accompanied by grain coarsening could produce a time exponent of 3.0 [26,27,28]. Görlich et al. [28] reported that intermetallic grains grew with a 1/3 power dependence on time only during the very early stages of the reaction, when grain coarsening of Ni_3_Sn_4_ IMCs actively occurred. Later, the kinetics changed to parabolic growth (n = 2), with the grain diameter fixed at a specific value. Furthermore, on electroless Ni-P metallization, the morphological change of the Ni_3_Sn_4_ IMCs, from needle to chunk, induced a high rate of grain coarsening. Consequently, the IMC growth rate declined according to the fast reduction of grain boundaries, resulting in a time exponent of 4.2 [29]. Based on these observations, IMC growth kinetics with a time exponent of 1.1–1.5 may not be attributed to grain boundary diffusion-controlled kinetics. Rather, the growth of the Ga_7_Ni_3_ IMCs in this study may be closely related to the interfacial reaction-controlled kinetics.

**Figure 10:**
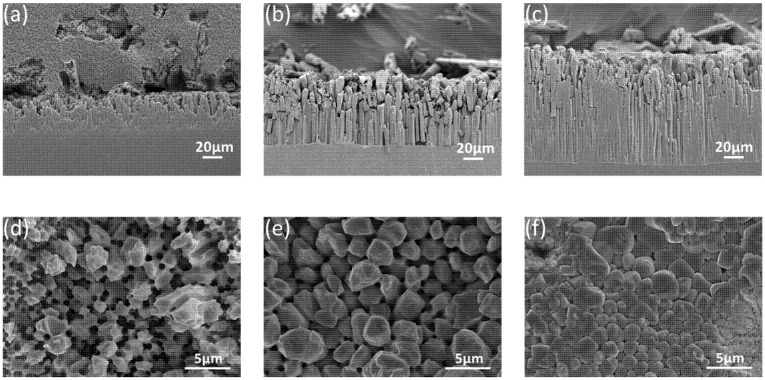
Cross-sectional SEM micrographs of interfacial Ga_7_Ni_3_ IMCs after the deep etching of Ga: (**a**) 30 min at 300 °C, (**b**) 60 min at 350 °C, (**c**) 90 min at 350 °C, and plane-view SEM micrographs of Ga_7_Ni_3_ IMCs, after complete etching of unreacted Ga: (**d**) 120 min at 250°C, (**e**) 120 min at 300 °C, (**f**) 120 min at 350 °C.

To further elucidate the IMC growth mechanism through more detailed studies of the IMC shape, cross-sectional SEM micrographs of interfacial Ga_7_Ni_3_ IMCs, after the deep etching of Ga, are presented in Figure 10a–c. The Ga_7_Ni_3_ IMCs formed during the reaction were long rods, and they grew only in a vertical direction with increasing reaction time. During their growth, no merging and no lateral coarsening of the IMC rods occurred. Plane-view SEM micrographs of the Ga_7_Ni_3_ IMCs, after the complete etching of unreacted Ga, are also presented in Figure 10d–f. The Ga_7_Ni_3_ IMC layers are observed to be very porous compared to the well-known Cu_6_Sn_5_ or Ni_3_Sn_4_ IMC layers, and the porosity decreases with increasing reaction temperature. When the reaction temperature was 250 °C, a large number of big holes were formed inside the IMC layer, whose diameters were approximately 0.6–1.0 μm. Large cavities occurred in regions where several holes were gathered closely. At 300 °C, the size and number of holes decreased. Most holes ranged from 0.5–0.8 μm in diameter. At 350 °C, small holes were observed around the triple points of the IMC rods. As the temperature increased, the individual IMCs became more distinct in shape, while the vacant areas between them became increasingly smaller.

Furthermore, the micrographs shown in Figure 10a–c confirmed that the observed holes reached the bottom of the IMC layer, forming deep channels between the Ga_7_Ni_3_ IMC rods. Unlike the grain boundaries between the Cu_6_Sn_5_ (or Ni_3_Sn_4_) grains, the channels between the Ga_7_Ni_3_ IMCs were filled with liquid Ga during the reactions. The Ni atoms dissolved into the channels are abundantly available around the reaction interface throughout the reaction time. The interface reaction-controlled growth kinetics (n = 1.1–1.5) for Ga_7_Ni_3_ IMCs may be attributed to the reaction environment, in which long-range diffusion of the atoms is not required for the growth of Ga_7_Ni_3_ IMCs. The channels decreased as the reaction temperature increased. At 250 °C, owing to the abundance of the atoms that crystallize into the Ga_7_Ni_3_ phase, the time exponent measured was very close to 1.0. On the other hand, at higher temperatures of 300 and 350 °C, fewer atoms would be available in the channels and, therefore, the diffusion length would be longer as the channels shrink, leading to larger time exponents of 1.37 and 1.50.

The activation energy E, for Ga_7_Ni_3_ growth was determined as 49.1 kJ/mol. Lin et al. [17] reported that E for Ga_2_Cu growth at temperatures of 200–240 °C was 23.8 kJ/mol. In their research, the time exponents were 1.0 for 160 °C and ~2.0 for the reactions at 200–240 °C, respectively. Therefore, the activation energies reported were for diffusion-controlled reactions, not for interface reaction-controlled kinetics. In comparison with the well-known Cu-Sn IMC growth, E for Ga_7_Ni_3_ in this study is located between the values of E for Cu_6_Sn_5_ (E = 19.7 kJ/mol) and Cu_3_Sn (E = 84.6 kJ/mol) [30].

## 4. Conclusions

In this study, the formation and growth of IMCs during the reaction between liquid Ga and nickel substrates were investigated. Ga and Ni were reacted at 250, 300, and 350 °C for 10 to 240 min. The resulting phase formation and IMC growth mechanism are summarized below.

(1) The IMC double layer was observed after the reactions consisted of a Ga_7_Ni_3_ bottom layer formed during the reaction, and a Ga_x_Ni top layer which was precipitated during the cooling process. The Ga_7_Ni_3_ layers were thickened with increasing reaction time, while the Ga_x_Ni layers were formed with a similar thickness of ~10 μm for all specimens reacted.
(2) The Ga-rich Ga_x_Ni phase possessed about 89–95 at.% of Ga, larger than the 83.3 at.% of Ga_5_Ni reported. This was not detected by XRD analysis, suggesting that the microstructure may not be perfectly crystalline, but nanocrystalline, or amorphous.
(3) The Ga_7_Ni_3_ layers were generally porous, and consisted of rod-type IMCs with empty holes between them. These empty channels were filled with liquid Ga during the reactions, into which the Ni atoms diffused, possibly playing an important role in Ga_7_Ni_3_ growth.
(4) The time exponents for Ga_7_Ni_3_ growth were estimated to be 1.1–1.5, which is thought to be governed by interface reaction-controlled kinetics supported by short-range diffusion. The activation energy for Ga_7_Ni_3_ growth was determined as 49.1 KJ/mol.

## Figures and Tables

**Table 1:** Phase formation and IMC growth kinetics reported in previous studies.

System	Temperature (°C)	IMC	Time Exponent (1/n)	Activation Energy (kJ/mol)	Reference
Cu/Ga	160	CuGa_2_ (+Cu_9_Ga_4_)	1.00	-	[17]
	200, 220, 240	CuGa_2_ (+Cu_9_Ga_4_)	0.50, 0.47, 0.54	23.8	
	300	Cu_9_Ga_4_	0.31	-	
Cu/Ga	350, 500	Cu_9_Ga_4_	~0.5	-	[19]
Ni/Ga	200, 350	Ga_7_Ni_3_	~0.5	-	
	500	Ga_3_Ni_2_	~0.5	-	
Cu/Ga/Ni	350	Cu_9_Ga_4_, Ga_7_Ni_3_	~0.5	-	
	500	Cu_9_Ga_4_, Ga_3_Ni_2_	~0.5	-	
Ni/Ga	300	Ga_7_Ni_3_ (+Ga_3_Ni_2_)	1.004	-	[20]
* Ni/Ni/Ga	300	Ga_7_Ni_3_ (+Ga_3_Ni_2_)	1.007	-	
Cu/Ni/Ga	300	Ga_7_Ni_3_ (+Ga_3_Ni_2_)	0.739	-	
Ni/Ga	250, 300, 350	Ga_7_Ni_3_	0.88, 0.67, 0.73	49.1	this study

* Substrate: electroplated Ni on bulk Ni.

## Data Availability

Not applicable.

## Supplementary Materials

The following are available online at https://www.mdpi.com/article/10.3390/ma14195694/s1, Figure S1: EDX analysis of the interfacial IMCs in the specimen reacted at 300 °C for 180 min; Figure S2: EDX analysis of the interfacial IMCs in the specimen reacted at 300 °C for 240 min; Figure S3: EDX analysis of the interfacial IMCs in the specimen reacted at 350 °C for 60 min; Figure S4: EDX analysis of the interfacial IMCs in the specimen reacted at 350 °C for 240 min.
