# Supplementary material for: Formation and Growth of Intermetallic Compounds during Reactions between Liquid Gallium and Solid Nickel
Source: Materials (Basel). 2021 Sep 30;14(19):5694. doi: 10.3390/ma14195694 (PMC8510494; doi:10.3390/ma14195694)
Supplement: Supplementary file 1 [file materials-14-05694-s001.zip › materials-1386677-supplementary.pdf]

materials

Supplementary Materials

Formation and Growth of Intermetallic Compounds during Reactions between Liquid Gallium and Solid Nickel

Doyoung Lee [1,†], Chang-Lae Kim [2,†] and Yoonchul Sohn [1,*]

[1] Department of Welding and Joining Science Engineering, Chosun University, Gwangju, 61452, South Korea; ldy6744@naver.com, yoonchul.son@chosun.ac.kr
[2] Department of Mechanical Engineering, Chosun University, Gwangju, 61452, South Korea; kimcl@chosun.ac.kr
† These authors contributed equally to this work.
* Correspondence: yoonchul.son@chosun.ac.kr

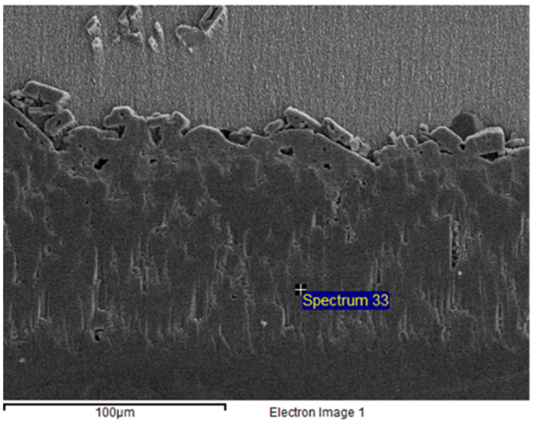

Element	Weight%	Atomic%
Ni K	29.65	33.36
Ga L	70.35	66.64
Totals	100.00	

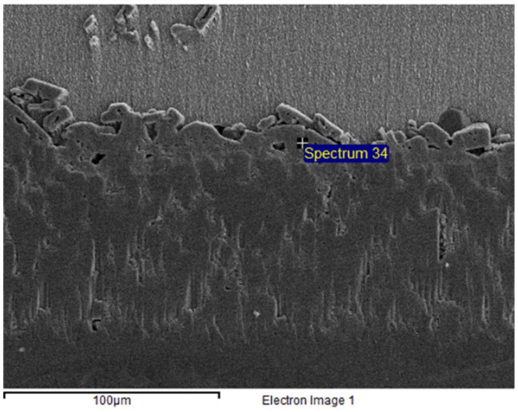

Element	Weight%	Atomic%
Ni K	9.33	10.89
Ga L	90.67	89.11
Totals	100.00	

Figure S1. EDX analysis of the interfacial IMCs in the specimen reacted at 300 °C for 180 min.

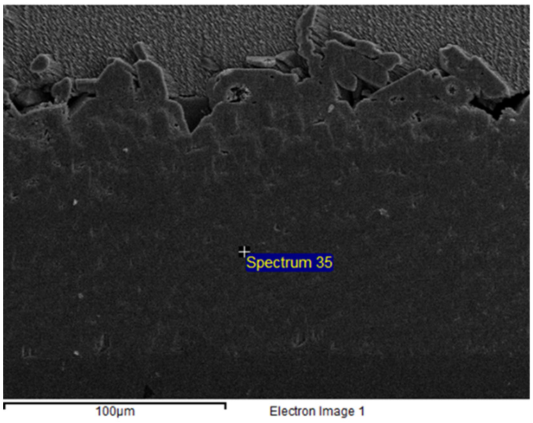

Element	Weight%	Atomic%
Ni K	26.79	30.29
Ga L	73.21	69.71
Totals	100.00	

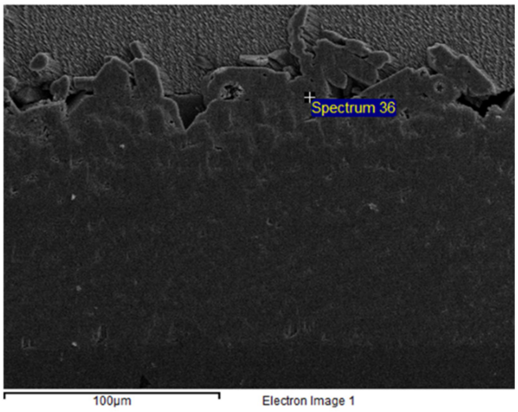

Element	Weight%	Atomic%
Ni K	4.23	4.99
Ga L	95.77	95.01
Totals	100.00	

Figure S2. EDX analysis of the interfacial IMCs in the specimen reacted at 300 °C for 240 min.

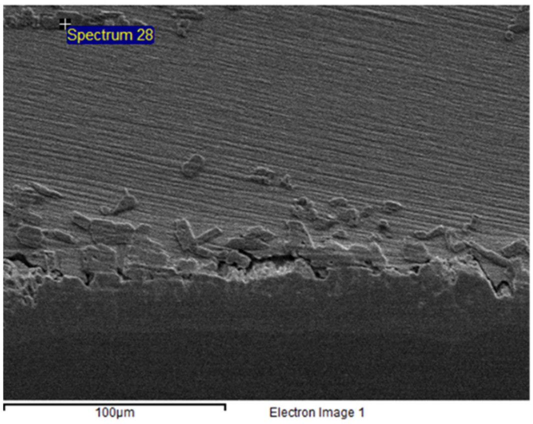

Element	Weight%	Atomic%
Ni K	6.79	7.96
Ga L	93.21	92.04
Totals	100.00	

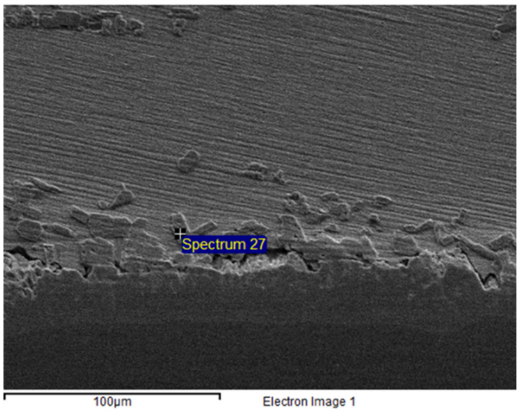

Element	Weight%	Atomic%
Ni K	6.32	7.42
Ga L	93.68	92.58
Totals	100.00	

Figure S3. EDX analysis of the interfacial IMCs in the specimen reacted at 350 °C for 60 min.

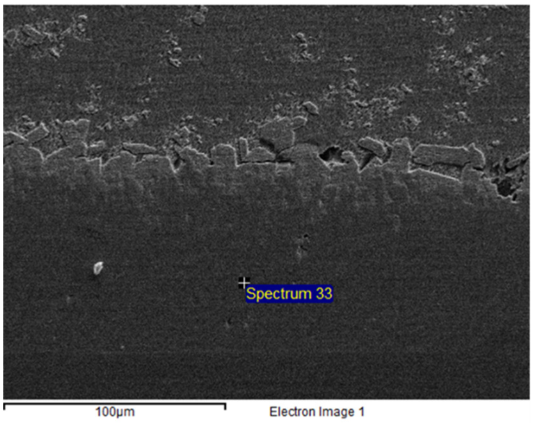

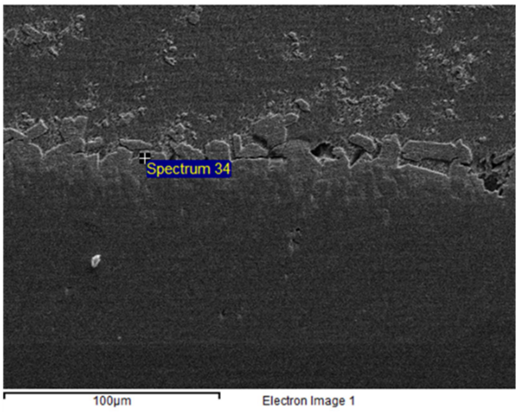

Element	Weight%	Atomic%
Ni K	25.65	29.06
Ga L	74.35	70.94
Totals	100.00	

Element	Weight%	Atomic%
Ni K	8.23	9.62
Ga L	91.77	90.38
Totals	100.00	

Figure S4. EDX analysis of the interfacial IMCs in the specimen reacted at 350 °C for 240 min.
